# Chronic Dysuria Following Ginger (*Zingiber officinale*) Use: A Case Report

**DOI:** 10.22086/gmj.v0i0.1086

**Published:** 2018-02-05

**Authors:** Elham Akbarzadeh, Mojtaba Heydari, Fatemeh Atarzadeh, Amir Mohammad Jaladat

**Affiliations:** ^1^Department of Traditional Persian Medicine, School of Medicine, Shiraz University of Medical Sciences, Shiraz, Iran

**Keywords:** Ginger, Dysuria, Case Report, *Zingiber Officinale*

## Abstract

**Background:**

Although ginger is considered a harmless remedial substance for a wide range of medical complaints, according to Persian medicinal texts, its long-term or high-dose consumption is potentially harmful.

**Case report:**

The case of a 43-year-old man, with a complaint of urinary stream interruption, dysuria, and flank pain, following a non-prescribed use of ginger was reported. The symptoms were reported to persist for four years, despite some medical referrals. Remarkably, the symptoms were attested to be shrinking eight weeks after ginger-intake cessation; besides, no further intervention was asserted.

**Conclusion:**

The history of herbal remedies use should be considered in patients with any unexplained urinary symptoms.

## Introduction

Although complementary and alternative medicine (CAM) practices are commonly applied [1], it seems that healthcare professionals and CAM product providers are not knowledgeable enough [2]. Furthermore, in this regard, the fact that self-medication mostly benefits from herbs/CAM should not be neglected; it should, however, also be noted that the rarity of reports covering the adverse effects of herbs could be secondary to their poor documentation and the absence of enquiries about patients’ use of herbal products [3].

As a final point, the dissemination of the adverse effects of the intake of wayward herbs_essential for establishing guidelines so as to integrate complementary and conventional therapies_is unquestionably strengthened and can be used in nursing education. In this report, we present a patient with chronic pain and dysuria, who was not advised regarding ginger consumption during the years of conflict.

## Case Presentation

A 43-year-old man claimed to suffer from interrupted urinary stream associated with dysuria, perineal, and flank pain for four years and referred to the Traditional Medicine Clinic affiliated with Shiraz University of Medical Sciences. Till that point, no improvement was reported during his frequent referrals to medical centers or in antibiotic therapies, such as ciprofloxacin and cefixime for sinusitis and upper respiratory infection. Physical examinations did not indicate any remarkable evidence.

Moreover, normal paraclinical investigations, including urine analysis, culture, prostatic specific antigen, and urinary system sonography were reported, so urinary tract infection, and other differential diagnoses like sexually transmitted diseases, obstructing urinary calculus, and prostatic abnormalities were rolled out. Although the patient had a feeling of partial seminal ejection during intercourse, he had experienced neither urinary frequency nor urgency.

Further, investigations of the history uncovered the feeling of warmness, chest heaviness, and palpitation for four years. Unexpectedly enough, more history analysis revealed that the patient—as a self-prescribed remedy in order to avert his knee-joint pain after prior old knee trauma—was consuming ginger tea (with a dosage of 2–3 teaspoons of dry ginger per day) for 15 years. The patient was obliged to stop taking ginger. Subsequently, within one week, the symptoms began to recede, and a complete disappearance was demonstrated after eight weeks without any further intervention. Also, no relapse in a six-month follow-up period was recorded.

## Discussion

Ginger (*Zingiber officinale* Roscoe) is globally used as a spice, and it is a popular medicinal root. Several studies have shown its effectiveness against nausea, inflammation, osteoarthritis symptoms, dysmenorrhea, hypercholesterolemia, memory improvement, and infertility. Even though it is commonly considered a harmless spice in dietary consumption, several side effects in its medicinal use have been reported in spontaneous reporting systems. The most predominant complaints include gastrointestinal symptoms followed by allergic reactions; ginger is regarded as a major contributor to these symptoms. Furthermore, cases of coagulation disorder, syncope, subacute thyroiditis, and electrolyte imbalance have also been documented in the literature [4, 5].

Furthermore, in traditional Persian medicine, ginger is believed to possess a potent “hot” nature alongside likely side effects, such as mucosal irritation culminating in dysuria (when consumed in high doses or after long-term use) [6].

The pathologic mechanisms that may link ginger consumption and dysuria include bladder epithelial dysfunction and peripheral and/or central neural upregulation. It has been suggested that other than stopping the intake spicy foods, elimination of sweet and sour tasting things from one’s diet (like citrus fruits and artificial sweeteners) may play an important role in the management of irritative urinary symptoms in patients with interstitial cystitis [7].

On the other hand, mucilaginous herbal drugs have been introduced for the treatment of dysuria, which is secondary to the urethral-moisturizing layer defect [8].

## Conclusion

Considering the present study’s findings accompanied by previous reports on the side effects of spicy foods consumption in the urinary system, consideration of patient’s history on ginger use can be of great importance.

## Conflicts of Interest

There are no conflicts of interest.
